# Epigenetic Aging Signatures in People with Hemophilia

**DOI:** 10.1055/a-2713-2910

**Published:** 2025-10-23

**Authors:** Daniel Kraemmer, Rafaela Vostatek, Marina Trappl, Johanna Gebhart, Ingrid Pabinger, Cihan Ay

**Affiliations:** 1 Division of Hematology and Hemostaseology, Department of Medicine I, Medical University of Vienna, Vienna, Austria

**Keywords:** hemophilia, aging, biological clocks, DNA methylation, CpG islands

## Abstract

**Introduction:**

Hemophilia is a rare X-linked bleeding disorder leading to recurrent hemarthroses, hemophilic arthropathy, and impaired quality of life. A chronic lifelong disease, hemophilia might be associated with accelerated biological aging. Here, we investigated whether biological age derived from epigenetic age estimators differs in hemophilia.

**Patients/Methods:**

We collected blood samples from men with severe (<1 IU/dL; PWSH, *n* = 20) or mild hemophilia (≥5 IU/dL; PWMH, *n* = 20), and age-matched healthy male controls ( *n* = 20). DNA methylation of cytosine–phosphate–guanine (CpG) dinucleotides at five genes ( *ASPA* , *ITGA2B* , *PDE4C* , *FHL2* , *CCDC10SB* ) was measured by bisulfite pyrosequencing. Biological age was estimated using two epigenetic aging signatures, each including three CpGs. We investigated differences in biological age and the rate of biological aging between study groups using separate linear regressions on chronological age and study group without and with an interaction, respectively.

**Results:**

Deviations of epigenetic from chronological age were high for both 3-CpG age estimators, with results suggesting systematic overprediction. In both linear regressions using the two 3-CpG estimates, respectively, evidence for a different rate of biological aging in severe hemophilia was weak. The rate of biological aging in PWSH was 0.24 (95% CI, 0.01–0.48) and 0.21 (0.04–0.37) higher compared with PWMH, and 0.05 (−0.19–0.29) and 0.17 (−0.00–0.34) higher compared with healthy controls, respectively. Hemophilic arthropathy was associated with an increased rate of biological aging.

**Conclusion:**

Evidence for a difference in epigenetic aging as reflected by two 3-CpG estimators in severe compared with mild hemophilia or healthy controls was weak.

## Introduction

Hemophilia A and B are rare congenital bleeding disorders caused by X-chromosomal mutations in the *F8* and *F9* genes, leading to deficiencies in clotting factor VIII (FVIII) and IX (FIX), respectively. 1 The clinical phenotype broadly correlates with the amount of residual endogenous factor activity, with factor activity <1 IU/dL commonly classified as severe, 1 to 5 IU/dL as moderate, and >5 to <40 IU/dL as mild hemophilia. 2 3 The hallmark of severe hemophilia is recurrent and spontaneous hemarthroses, which cause synovitis and damage to the articular cartilage and bone, eventually leading to disabling hemophilic arthropathy of the affected joint. 4 With advancements in therapeutic options and early initiation of regular lifelong prophylaxis, the life expectancy of people with hemophilia, while still impaired, has significantly increased in recent decades, in particular in high-income countries. 5 Nevertheless, lacking a definite curative therapeutic option, hemophilia remains a chronic disease with impaired joint health and substantial limitations in quality of life, such as acute and chronic pain, interference in daily life and physical activity, and increased obesity, depression, and anxiety. 6 7 8 New developments, such as non-factor therapies, ultralong extended half-life concentrates, and gene therapy, raise the prospect of further narrowing this gap between people with hemophilia and the general population. 9 10 11

Biological age has been proposed as a conceptual value that might better reflect the extent of aging-driven biological changes than chronological age, merely measuring calendar time that has passed since birth. 12 13 Many chronic diseases are expected to affect such a biological age estimate, and several biomarkers have been studied to date. 14 15 16 One such attempt revolves around measuring epigenetic alteration in the form of cytosine methylation in cytosine–phosphate–guanine (CpG) dinucleotides, that is, DNA regions where a cytosine nucleotide is followed by a guanine nucleotide. These methylation patterns at specific CpG sites have been shown to change with age. 17 Several epigenetic age estimators, often referred to as "epigenetic clocks", have been proposed. 18 19 20 21 22 23 First-generation epigenetic clocks typically consist of simple linear models including a set of CpG sites, which are selected based on their correlation with chronological age and use the weighted sum of their DNA methylation states to calculate an epigenetic age estimate. Their deviations from chronological age have been associated with age-related conditions and all-cause mortality and, thus, have been suggested as a potential surrogate marker for biological age. 17 In 2014, Weidner and colleagues published a simple epigenetic aging signature model to estimate the state of aging in blood by measuring DNA methylation of just three CpG sites. 24

Here, we aimed to investigate the difference in biological aging as derived from such epigenetic age estimators between people with severe compared with mild hemophilia and age- and sex-matched healthy controls.

## Materials and Methods

### Blood Samples

All male people with severe (PWSH; FVIII/FIX <1 IU/dL) or mild hemophilia A or B (PWMH; FVIII/FIX >5 IU/dL) participating in our hospital-based biobank on patients with inherited bleeding disorders (EC 981/2011) were eligible for study inclusion. We selected blood samples from 20 PWSH and 20 PWMH, chosen to ensure a broad coverage of the age range represented in our biobank. In a second step, we further collected samples from 20 male healthy controls, matched by chronological age (±5 years) to the whole cohort of people with hemophilia (EC 039/2006). All participants gave their written informed consent for inclusion. The study had approval from the local ethics committee and was conducted in accordance with the ethical principles set forth in the Declaration of Helsinki.

### DNA Methylation

Blood was sampled from an antecubital vein with a 21-gauge butterfly needle into an EDTA-coated blood collection tube. Aliquots of whole blood samples were stored at −20 °C until further analysis. DNA methylation analysis at age-associated CpG sites was externally analyzed as described previously, with external analysts blinded to any patient characteristics. 24 In brief, genomic DNA was isolated from 200 μL of blood using the QIAamp DNA Mini Kit (Qiagen, Hilden, Germany). DNA concentration was quantified by Nanodrop 2000 Spectrophotometers (Thermo Scientific, Wilmington), and 200 ng of genomic DNA was subsequently bisulfite-converted with the EZ DNA Methylation Kit (Zymo Research, Irvine) and subjected to PCR amplification within the genes *ITGA2B* , *ASPA* , and *PDE4C* . In addition, three CpGs in the genes *FHL2* , *CCDC102B* , and *PDE4C* were analyzed as described previously. 25 Amplicons were sequenced on the PyroMark Q48 System (Qiagen, Hilden, Germany) and analyzed with the PyroMark Q CpG software (Qiagen).

### Epigenetic Age Estimation

Epigenetic age was estimated from two epigenetic aging signatures (3-CpG estimators).

**3-CpG estimator 1** was based on DNA methylation at three CpGs in the genes *ASPA* , *ITGA2B* , and *PDE4C* , as published by Weidner and colleagues (α = *ASPA* , β = *ITGA2B* , γ = *PDE4C* ). 24



**3-CpG estimator 2** was based on the DNA methylation levels at three CpGs in the genes *FHL2* , *CCDC102B* , and *PDE4C* , which had been selected on a larger set of available DNA methylation profiles, as described previously (δ = *FHL2* , ε = *CCDC102B* , γ = *PDE4C* ). 25



### Statistics

To investigate differences between the three study cohorts, we fitted linear models using ordinary least squares, regressing biological age as calculated from the 3-CpG estimators on chronological age and study group, first comparing people with hemophilia to healthy controls and, subsequently, differentiating between severe and mild hemophilia. By including an interaction between chronological age and study cohort, we allowed for different slopes between groups, that is, different rates of change in epigenetic age, instead of assuming the difference in biological aging between groups to be constant over time (as would be the case in models lacking an interaction). Evidence for a difference in rates between groups was expressed by reporting *p* -values from partial F-tests comparing the regression model including the interaction with the nested model without the interaction. All statistical analyses were conducted in R (version 4.4.2). 26

## Results

**Fig. 1:**
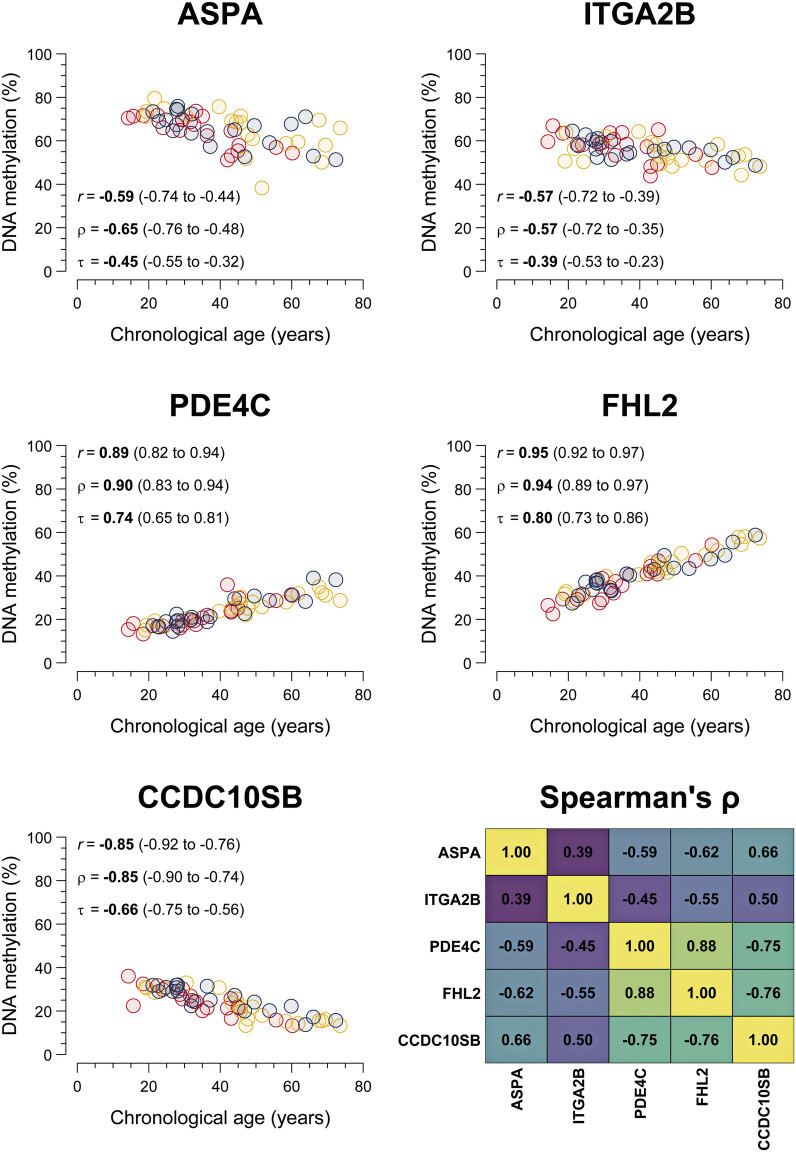
Correlations of DNA methylation of the five investigated genes with chronological age. Scatter plots comparing DNA methylation (in percentage) of the five investigated CpG sites (in the genes *ASPA* , *ITGA2B* , *PDE4C* , *FLH2* , and *CCDC10SB* ) with chronological age, stratified by cohort group (severe hemophilia, red; mild hemophilia, yellow; healthy controls, blue). The heatmap displays Spearman's rank correlation coefficients of the DNA methylation between the different CpG sites. 3-CpG age estimator 1 included CpG DNA methylation at the genes *ASPA* , *ITGA2B* , and *PDE4C* ; and the 3-CpG age estimator 2 included CpG sites at *PDE4C* , *FHL2* , and *CCDC10SB* . Pearson's correlation coefficient ( *r* ), Spearman's rank correlation coefficient (ρ), and Kendall's rank correlation coefficient (τ) with 95% percentile bootstrap confidence intervals in parentheses quantify the correlations of the respective CpG DNA methylation and chronological age. CpG, cytosine–phosphate–guanine.

Characteristics of people with hemophilia and the control group are shown in Table 1 . Age, and body mass indices were higher in the mild hemophilia group (hemophilia A, *n* = 17/20) compared with subjects with severe hemophilia (hemophilia A, *n* = 19/20) and healthy controls. Chronological age showed an inverse ( *ASPA* , *ITGA2B* , *CCDC102B* ) or positive ( *PDE4C* , *FHL2* ) association with DNA methylation levels at the five investigated CpG sites, although evidence for a difference by study group was lacking ( Fig. 1 ).

**Table 1:** Descriptive summary statistics of the people with severe hemophilia, mild hemophilia, and healthy controls

	Hemophilia ( *N* = 40)	Severe hemophilia ( *N* = 20)	Mild hemophilia ( *N* = 20)	Healthy controls ( *N* = 20)
Clinical characteristics				
Hemophilia A	35	18 (0.90)	17 (0.85)	0 (0.0)
Hemophilia B	5	2 (0.10)	3 (0.15)	0 (0.0)
Male sex	40 (1.00)	20 (1.00)	20 (1.00)	20 (1.00)
Chronological age (years)	40.3 ± 15.9 (14.3, 73.6)	34.3 ± 12.4 (14.3, 60.2)	46.4 ± 17.0 (19.1, 73.6)	40.1 ± 15.9(21.1, 72.3)
Body mass index (kg/m ^2^ )	25.0 ± 3.9	24.1 ± 3.7	26.0 ± 4.0	23.8 ± 3.1
Hemophilic arthropathy	17 (0.42)	16 (0.80)	1 (0.05)	0 (0.00)
Hepatitis C	15 (0.38)	9 (0.45)	6 (0.30)	0 (0.00)
HIV	4 (0.10)	4 (0.14)	0 (0.00)	0 (0.00)
DNA methylation				
*ASPA* (%)	68.0 (58.6–71.3)	66.8 (57.6–70.7)	68.5 (59.3–71.3)	67.2 (62.4–71.4)
*ITGA2B* (%)	56.6 (50.8–59.3)	58.3 (53.6–61.4)	52.9 (50.5–58.4)	56.2 (53.5–59.6)
*PDE4C* (%)	23.6 (18.0–28.1)	19.5 (17.2–24.2)	25.9 (21.6–29.1)	21.1 (18.9–28.9)
*FHL2* (%)	40.9 (32.0–46.5)	36.5 (29.3–41.2)	45.0 (40.4–50.7)	39.3 (35.9–44.6)
*CCDC10SB* (%)	22.4 (16.6–28.9)	24.3 (21.4–28.2)	20.9 (15.5–29.0)	26.1 (21.7–30.8)
Estimated epigenetic age				
3-CpG age estimator 1 (years)	47.2 (34.9–56.6)	37.5 (34.4–51.6)	51.0 (41.0–58.2)	42.2 (36.1–56.3)
3-CpG age estimator 2 (years)	48.7 (30.9–59.3)	38.9 (29.4–51.6)	56.1 (41.7–66.5)	39.0 (35.9–56.8)

Abbreviations: CpG, cytosine–phosphate–guanine; HIV, human immunodeficiency virus; Q, quartile; SD, standard deviation.

Frequency (proportion); mean ± SD; median (Q1-Q2); (minimum, maximum).

### 3-CpG Estimator 1

**Fig. 2:**
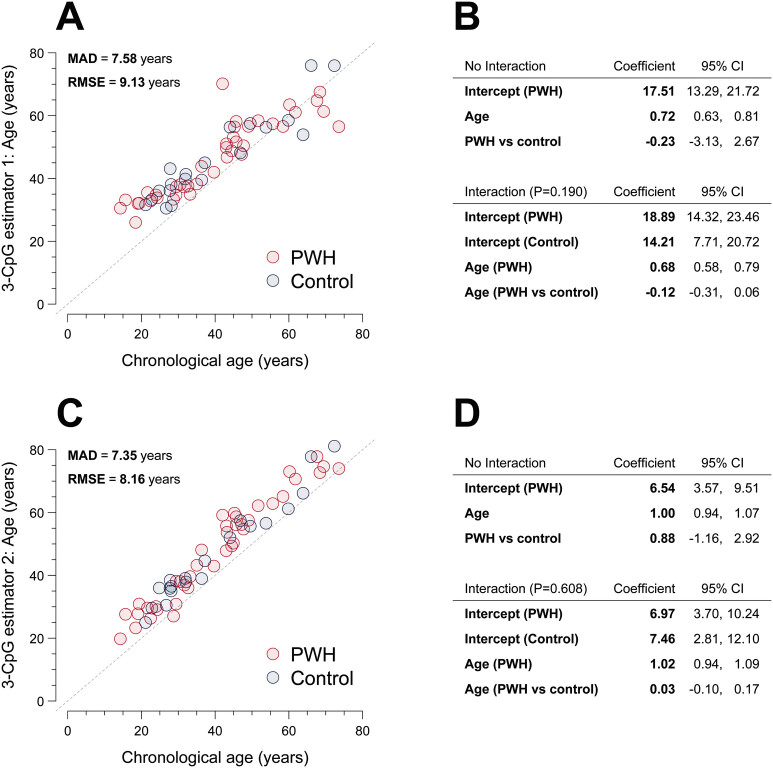
Comparison of estimated epigenetic age between people with hemophilia and healthy controls. ( **A, C** ) display scatter plots of the epigenetic age derived from 3-CpG age estimators 1 and 2, respectively, with chronological age. People with hemophilia (PWH) and healthy controls are depicted in red and blue, respectively. ( **B, D** ) show the coefficients and 95% confidence intervals (CIs) from linear regression of the respective epigenetic age estimate on chronological age (abbreviated “Age”) and PWH/control. P denotes *p* -values from a partial F-test comparing the model with an interaction to the respective nested model without the interaction. CI, confidence interval; CpG, cytosine–phosphate–guanine; MAD, mean absolute deviation; PWH, people with hemophilia; RMSE, root mean squared error; vs, versus.

**Fig. 3:**
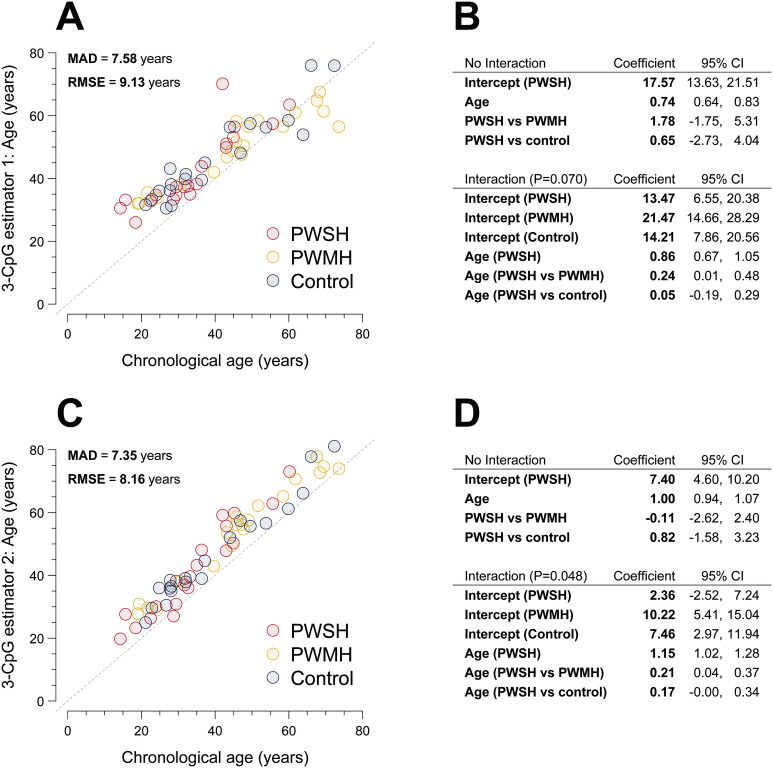
Comparison of estimated epigenetic age between people with hemophilia stratified by severity and healthy controls. ( **A, C** ) display scatter plots of the epigenetic age derived from 3-CpG age estimators 1 and 2, respectively, with chronological age. People with severe hemophilia (PWSH), people with mild hemophilia (PWMH), and healthy controls are depicted in red, yellow, and blue, respectively. ( **B, D** ) show the coefficients and 95% confidence intervals (CIs) from linear regression of the respective epigenetic age estimate on chronological age (abbreviated “Age”) and study group. P denotes *p* -values from a partial F-test comparing the model with an interaction to the respective nested model without the interaction. CI, confidence interval; CpG, cytosine–phosphate–guanine; MAD, mean absolute deviation; PWMH, people with mild hemophilia; PWSH, people with severe hemophilia; RMSE, root mean squared error; vs, versus.

In the subset of healthy controls ( *n* = 20, 33.3%), epigenetic age as estimated by 3-CpG estimator 1 had a mean absolute deviation (MAD) from chronological age of 7.42 years, exceeding the MAD values in the training (5.43 years) and validation cohort (4.49 years) reported by Weidner and colleagues. 24 The MAD for all samples was 7.58 years ( Fig. 2A ). Notably, the estimated epigenetic age exceeded the chronological age in all subjects aged <58 years ( *n* = 49, 81.7%). In linear regression of biological on chronological age, model-predicted mean epigenetic age increased slower than chronological age, that is, by 0.72 years (95% CI, 0.63–0.81) for every increase in chronological age, which was offset by a very high intercept, reflecting the relative overprediction in all subjects <58 years. Estimated differences in mean epigenetic age between people with hemophilia and healthy controls were inconclusive ( Fig. 2B ). Our data were compatible with both a lower and higher mean epigenetic age in PWSH compared with PWMH and healthy controls, with differences ranging from −1.75 to 5.31 and from −2.74 to 4.04 years, respectively ( Fig. 3A, B ). The evidence for an interaction between chronological age and group, that is, a difference in the rate by which epigenetic age changed with increasing chronological age between study groups, was weak ( *p* -value = 0.070), as was the evidence for a higher rate among PWSH compared with healthy controls (0.05; 95% CI, −0.19–0.29) or PWMH (0.24; 95% CI, 0.01–0.48).

### 3-CpG Estimator 2

The MAD for the 3-CpG estimator 2 was 6.71 and 7.35 years in the subset of healthy controls and all samples, respectively ( Fig. 2C ). Except for one subject with severe hemophilia, all study participants ( *n* = 59, 98.3%) had their epigenetic age as calculated from 3-CpG estimator 2 exceeding their chronological age. In linear regression, mean epigenetic age increased at a similar rate as chronological age, that is, by 1.00 (95% CI, 0.94–1.07) years for every increase in chronological age ( Fig. 2D ). The systematic overprediction was reflected by an intercept of 6.54 years (95% CI, 3.57–9.51), resulting in the observed shift above the line of equivalence. Again, evidence for a difference in epigenetic age or a difference in the rate of change between people with hemophilia and healthy controls was lacking. We found limited evidence ( *p* -value = 0.048) for an interaction indicating different changes in epigenetic age by study group as chronological age increased. In PWSH, the rate at which mean epigenetic age increased by chronological age was higher by 0.17 (95% CI, 0.00–0.34) and 0.21 (95% CI, 0.04-0.37) compared with healthy controls and PWMH, respectively, although our data were compatible with negligible differences ( Fig. 3C, D ).

### Subgroup Analyses

**Fig. 4:**
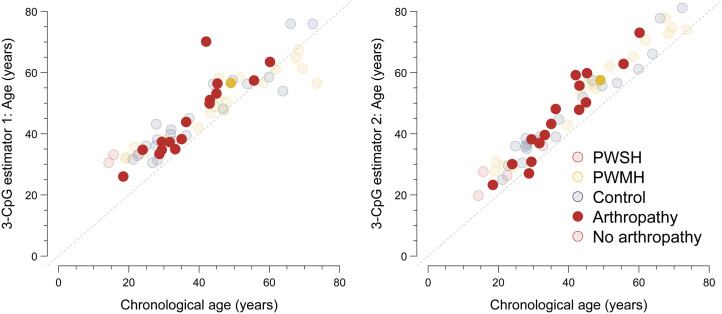
Samples from subjects with hemophilic arthropathy. Scatter plots of the epigenetic age estimates derived from 3-CpG age estimators 1 and 2, respectively, with chronological age, stratified by study group. People with severe hemophilia (PWSH), people with mild hemophilia (PWMH), and healthy controls are depicted in red, yellow, and blue, respectively. Transparent points indicate absence and solid points the presence of hemophilic arthropathy. CpG, cytosine–phosphate–guanine; PWMH, people with mild hemophilia; PWSH, people with severe hemophilia.

As expected, hemophilic arthropathy was present in a far higher proportion of PWSH ( *n* = 16, 80.0%) than PWMH ( *n* = 1, 5.0%; Fig. 4 ). Stratifying all samples by presence of hemophilia arthropathy, we found evidence suggesting an increased rate of biological aging as derived from CpG estimator 1 (0.30; 95% CI, 0.05–0.54) and CpG estimator 2 (0.24; 95% CI, 0.07–0.41), respectively.

## Discussion

In this study, we investigated whether biological age as estimated by 3-CpG age estimators differed between severe compared with mild hemophilia and healthy controls. Evidence for a higher mean biological age or higher rate of biological aging in severe hemophilia was weak, with our results being compatible with no or negligible differences between groups. We did, however, find evidence suggesting an increased rate of biological aging in those subjects with hemophilic arthropathy.

DNA methylation-based age estimation has been investigated in a variety of age-related conditions and diseases. It has been suggested as a potential aid in augmenting risk prediction in cardiovascular disease and mortality. However, data on bleeding disorders or hemophilia are lacking. 17 27 Conceptually, hemophilia, particularly severe hemophilia, might be associated with increased biological age due to chronic low-grade inflammation, which has been described previously and could impact joint health, tissue regeneration, and cardiovascular disease. 28 Recurrent hemarthroses not only lead to chronic joint inflammation and eventually hemophilic arthropathy but also impair quality of life and participation in physical activity, thereby possibly predisposing to depression, obesity, a sedentary lifestyle, and cardiovascular comorbidities. 6 7 8 The occurrence of the latter, when necessitating antithrombotic treatment, poses substantial challenges complicating adequate therapy, particularly in severe hemophilia. 29 Furthermore, liver disease and viral infections, such as HIV and hepatitis, remain an important burden in the older hemophilia population. 30

To our knowledge, epigenetic age as estimated by DNA methylation patterns at CpG sites has not been investigated in hemophilia. In this study, we did not find convincing evidence for a difference in epigenetic age between hemophilia and healthy controls. Several other models and biomarkers have been proposed for estimating biological age. Two such alternative biomarkers are telomere length and mitochondrial DNA copy number, both of which have been suggested to be associated with biological age. 31 Recently, we compared telomere length and mitochondrial DNA copy number in people with severe and mild hemophilia to healthy controls and found a significant decrease of both biomarkers in the hemophilia cohort, possibly indicating increased biological aging. 32 One notable difference in the method used in the present study is that CpG site selection and training of first-generation epigenetic age estimators are based on chronological age and, thus, might arguably reflect chronological age instead of epigenetic influences. This issue is demonstrated by the observation that increasing the training sample sizes of those estimators for increasingly perfect predictions of chronological age reduces their ability to detect variations in biological aging between individuals as the age gap between epigenetic and chronological age approaches zero. 33 In fact, clinical and lifestyle parameters showed substantial variability and only a weak association with age as predicted by the estimator used in this study. 24 While there has been some evidence for an association with all-cause mortality beyond established risk factors among first-generation epigenetic age estimators, associations have generally been weak and possibly negligible, further questioning whether they reflect epigenetic age. 34 35

There are several important limitations to our investigation that need to be considered in light of the apparent lack of evidence for a difference in biological aging between study groups. First and foremost, the low sample size hampered our ability to detect differences in biological aging, particularly the rate of biological aging, between study groups. Investigating the latter was of interest, as, conceptually, a chronic condition like hemophilia would be expected to exhibit time-dependent effects on biological age instead of a mere shift that is constant over the whole lifespan. Unfortunately, more granular subgroup analyses were severely limited. Finally, especially considering the high mortality following the widespread hepatitis C and HIV infections in older PWSH, survival bias might have severely confounded any effect of hemophilia on accelerated biological aging.

## Conclusion

We investigated biological age as predicted by two 3-CpG age estimators in hemophilia. Evidence for a difference in biological aging or accelerated biological aging in severe compared with mild hemophilia or healthy controls was weak. We found limited evidence for an association of hemophilic arthropathy with accelerated biological aging.
